# Prescribed cumulative dosage of corticosteroids to patients with inflammatory bowel disease diagnosed between 2006 and 2020: a retrospective observational study

**DOI:** 10.1177/17562848241288851

**Published:** 2024-10-13

**Authors:** Johannes Iiristo, Pontus Karling

**Affiliations:** Department of Public Health and Clinical Medicine, Umeå University, Umea, Sweden; Department of Public Health and Clinical Medicine, Umeå University, Umea, 901 87, Sweden

**Keywords:** 5-ASA, biologics, budesonide, Crohn’s disease, immunomodulators, inflammatory bowel disease, prednisolone, surgery, ulcerative colitis

## Abstract

**Background:**

Treatments and strategies for inflammatory bowel disease (IBD) have gradually evolved in the 2000s.

**Objectives:**

We investigated whether the prescription of corticosteroids (prednisolone and budesonide) in patients with IBD in the first 5 years after diagnosis changed in patients diagnosed between 2006 and 2018.

**Design:**

Retrospective observational study.

**Methods:**

The cumulative prescribed dosage of corticosteroids for the first 5 years after diagnosis was registered in all patients with IBD (*n* = 386) at our clinic for those diagnosed between 2006 and 2018.

**Results:**

The proportion of patients with IBD who were prescribed at least one prescription of corticosteroids in year 1–5 after diagnosis was 55.3%, 27.9%, 22.7%, 14.1%, and 14.6%, respectively. The proportion of patients who had a cumulative dose of prednisolone >1 g in the first 5 years after diagnosis was 40.1% for ulcerative colitis and 34.9% for Crohn’s disease (CD). The cumulative prescribed dosage (within 3 years after diagnosis) of prednisolone had declined (rs = −0.164, *p* = 001), but had increased for budesonide (rs = 0.202, *p* < 0.001) between 2006 and 2020. The prescription of any immunomodulator for IBD in the first 5 years from diagnosis was stable between 2006 and 2018 (rs = 0.056, *p* = 0.257), but there was a minor increase in the prescription of Tumor Necrosis Factor (TNF)-inhibitors (rs = 0.119, *p* = 0.020). The use of five-acetyl salicylic acid (5-ASA) decreased in patients with CD (rs = −201, *p* = 0.012).

**Conclusion:**

There was a decrease in the prescription of prednisolone and an increase in the prescription of budesonide treatment from 2006 to 2023; however, the cumulative exposure to corticosteroids in patients with IBD remains at a relatively high level.

## Plain language summary

The use of steroids in patients with inflammatory bowel disease diagnosed between 2006 and 2020

In the 1950s, corticosteroids and immunomodulators were introduced, and in combination with improved surgery, the mortality rates dramatically decreased in patients with inflammatory bowel disease (IBD) Although corticosteroids are effective in the short term they have no proven efficacy in long-term therapy for IBD, and owing to the risk of side effects, their long-term use should be restricted. Based on the evolution of treatments and treatment strategies for IBD in the 2000s, we aimed to study to the extent to which corticosteroids have been used in the first five years after diagnosis for patients with IBD diagnosed at our clinic between 2006 and 2020. To what extent is prednisolone prescribed in the first five years after diagnosis? Has the pattern of corticosteroid prescription changed after the introduction of advanced therapy and biosimilars to TNF inhibitors? We found that the proportion of patients with IBD who were prescribed at least one prescription of corticosteroids in year one to five after diagnosis was 55.3%, 27.9%, 22.7%, 14.1%, and 14.6%, respectively. The proportion of patients who had a cumulative dose of prednisolone >1g in the first five years after diagnosis was 40.1% for ulcerative colitis (UC) and 34.9% for Crohns disease (CD). The cumulative prescribed dosage (within 3 years after diagnosis) of prednisolone had declined but increased for budesonide between 2006-2020. The prescription of any immunomodulator for IBD in the first five years from diagnosis was stable between the years 2006-2018, but there was a minor increase in the prescription of TNF-inhibitors. The use of 5-ASA decreased in patients with CD. We conclude that there was a decrease in the prescription of prednisolone and an increase in the prescription of budesonide treatment from 2006 to 2023; however, the cumulative exposure to corticosteroids in patients with IBD remains on relatively high level.

## Introduction

In the 1950s, corticosteroids and immunomodulators were introduced, and in combination with improved surgery, the mortality rates dramatically decreased in patients with inflammatory bowel disease (IBD).^1,2^ Although corticosteroid treatment is effective in the short term it has no proven efficacy in maintenance therapy for IBD; and owing to the risk of side effects, its long-term use should be restricted.^3–6^ Therefore, “Steroid-free remission” is an established endpoint in modern clinical trials.^7,8^ However, in a large register study from the United States, approximately 6% of the patients with IBD were prescribed chronic corticosteroid therapy; and in that study, patients treated with corticosteroids had an increased risk of opportunistic infections, osteoporosis, thromboembolism, Cushing’s syndrome, and adrenal insufficiency.^9^ In a Danish registry study, patients with even a relatively low cumulative dose (>1 g prednisolone) had an increased risk of hip fractures.^10^

The introduction of biologics in 1998^11,12^ has led to the availability of other effective induction therapies for IBD. However, the use of biologics was initially restricted owing to their high cost. The first biosimilar for TNF inhibitors (infliximab) was approved in Europe in 2013, and soon after, biosimilars gradually replaced original TNF inhibitors, thus reducing the costs of medical treatment with TNF inhibitors.^13^ Concomitantly, newer biologics such as vedolizumab^14^ and ustekinumab^15^ were introduced as treatments for IBD, followed by new treatment strategies such as janus-kinases inhibitors (tofacitinib).^16^ With the development of IBD therapies, the use of corticosteroids has declined, but the extent varies between countries.^17–22^

Based on the evolution of treatments and treatment strategies for IBD in the 2000s, we explored the use of corticosteroids in the first 5 years following diagnosis of IBD, and whether the pattern of corticosteroid use has changed with the introduction of advanced therapy and the introduction of biosimilars to TNF inhibitors.

## Material and methods

### Study design

This quality research project was constructed as a retrospective observational study. The reporting of this study conforms to the Strengthening the Reporting of Observational Studies in Epidemiology (STROBE) statement.^23^

### Patients

**Figure 1.**
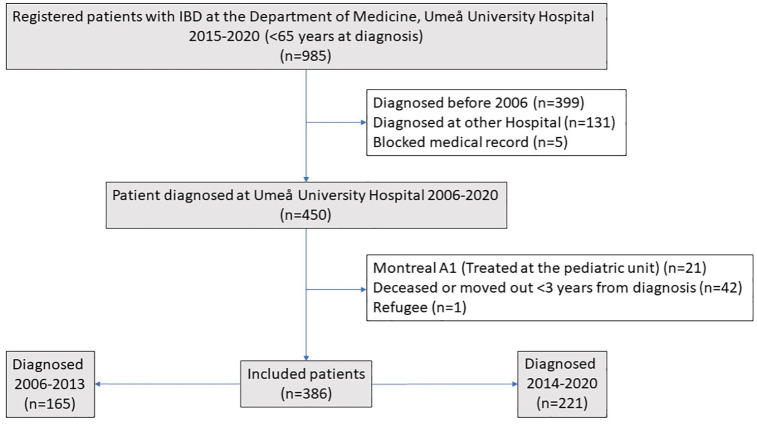
Flow chart showing the process of patient inclusion in the study.

All patients who visited the gastroenterology unit at the Department of Medicine at Umeå University Hospital and were diagnosed with Crohn’s disease (CD; ICD code K50.0-K50.9) or ulcerative colitis (UC; ICD code K51.0-K51.9) between 2015 and 2020 were identified. Umeå University Hospital is an academic center with a primary catchment area of approximately 150 000 citizens and is located in the northern part of Sweden. All patients with IBD in the region are diagnosed and treated at our hospital and all are coded with an ICD code at each visit. The study focused on patients diagnosed between 2006 and 2020, and the patients had to be diagnosed after the age of 17 years (Montreal classification^24^ A2 and A3) and before the age of 65 years. The date of diagnosis was defined as the first endoscopic or radiological investigation in which the patient showed signs of IBD. Patients who were diagnosed outside the catchment area or patients who moved out of the catchment area within 3 years of diagnosis were excluded. Figure 1 shows the flow chart of the patients included in the study.

### Data collection from medical records

All data in the study are based on information from our digital medical record system (NCS Cross), which includes data on health care visits, endoscopy, surgery, radiology, and data on all prescriptions of drugs in the Region of Västerbotten. The collected information is based on free text and from the drug portal within the digital medical record. All prescribed drugs in the region are visible in the drug portal, and for each prescription, information on dosage, amount, and treatment causes are usually presented.

### Treatment

The cumulative dose of prescribed prednisolone and budesonide was calculated from the date of diagnosis and yearly up until 5 years after diagnosis. When detailed information on dosage was missing, a course of prednisolone was estimated to be 980 mg (35 mg for 7 days, weaning down by 5 mg every 7 days for a total course of 7 weeks) and a course of budesonide was 540 mg (9 mg once daily for 30 days, weaned down by 3 mg every 30 days). Only corticosteroid treatments prescribed for IBD have been reported. A high dose of cumulative systemic prednisolone was defined as a cumulative dose >1 g, which is equivalent to >1 course of prednisolone. According to ECCO guidelines,^25,26^ a corticosteroid-sparing agent (immunomodulator or biologics) should be initiated for any patient who requires more than a single course of systemic corticosteroids in a year. In our study, we defined an indication to start a corticosteroid-sparing agent if a patient has prescribed a dosage of prednisolone >1 g within a year. For rectal administration of corticosteroids, we used a compound variable that included prednisolone and budesonide, irrespective of the type of rectal treatment. For surgery and for thiopurines, TNF inhibitors, and second-line treatment; the time to start of treatment was recorded. In the variable five-acetyl salicylic acid (5-ASA), both rectal and oral treatments were included. In our study, second-line treatment was defined as treatment with at least one of the drugs vedolizumab, ustekinumab, or tofacitinib.

### Statistical methods

The data were analyzed using IBM SPSS version 28.0, IBM corporation, New York, USA. The Chi-square test was used to compare proportions (Fisher exact test was used if the numbers were small). The Student’s *t* test was used to compare the mean age. The Mann–Whitney test was used to compare the median dosages of corticosteroids. Spearman’s test was used for the correlation analysis. Kaplan-Meier curves were used to illustrate the time to start of treatment, and for statistical analysis, the Cox–Mantel test, was used to compare time to start of treatments. Censoring was used to compensate for incomplete data and differences in follow-up data. To test trends in IBD treatment, we also divided the patients into two groups based on the year of diagnosis. The first group included patients diagnosed between 2006 and 2013 and the second group included patients diagnosed between 2014 and 2020 (Figure 1). We used the 2013 cut-off because biosimilars were introduced in Sweden after that year. To calculate the proportion of patients at 4 and 5 years respectively, only patients with data on these time points were included. For the 5-year follow-up data, only patients diagnosed between 2006 and 2018 were included. We did not adjust for multiple testing.

## Results

### Basal characteristics

Overall, 386 patients fulfilled the inclusion criteria (232 patients with UC and 154 patients with CD). The median age for the patients was 32 years (25th–75th percentile 25–43 years) and the proportion of women and men was about the same (48.4% women and 51.6% men). There were no significant differences in age at diagnosis, sex, or Montreal Classification in patients with UC diagnosed 2006–2013 versus those diagnosed 2014–2020 (Table 1).

**Table 1.** Patients with ulcerative colitis diagnosed 2006–2013 versus those diagnosed in 2014–2020.

	2006–2013 (*n* = 109)	2014–2020 (*n* = 123)	*p*-Value
Mean age at diagnosis, years (SD)	34.8 (11.7)	34.0 (12.6)	0.618
Proportion of women, %	52.3	48.8	0.593
Montreal classification, %			
A2 (17–40 years)	73.4	74.0	0.919
A3 (40–65 years)	26.6	26.0	0.919
E1 (proctitis)	26.6	32.5	0.325
E2 (left-sided colitis)	33.3	34.1	0.896
E3 (extensive colitis)	39.4	33.3	0.333
Prednisolone in the first 5 years since diagnosis^a^			
Any prescription, %	49.0	52.0	0.670
Median cumulative dose, mg (25th–75th percentile)	0 (0–2352)	565 (0–1540)	0.595
Proportion of patients with a cumulative prednisolone dose >1 g, % (*n* = 65/82)	46	34	0.139
Proportion of patients with a cumulative prednisolone dose >3 g, % (*n* = 64/82)	22	13	0.193
Budesonide in the first 5 years since diagnosis^a^			
Any prescription, %	4.8	13.1	0.066
Median cumulative dose, mg (25th–75th percentile)	0 (0–0)	0 (0–0)	0.039
Any prescription of rectal corticosteroids, %	58.7	37.4	0.001
Any prescription, %			
5-ASA	97.2	97.6	0.881
Thiopurines	33.9	32.5	0.818
TNF inhibitors	11.9	19.5	0.115
Vedolizumab	1.8	2.4	0.891
Ustekinumab	0	1.6	0.499
Tofacitinib	0	2.4	0.249
Colectomy within the first 5 years since diagnosis,^a^ %	4.6	0.8	0.103

a Includes only patients diagnosed 2006–2018.

5-ASA, five-acetyl salicylic acid.

Compared to patients with CD diagnosed in 2014–2020, patients with CD diagnosed in 2006–2013 had a significantly lower proportion of women, a higher proportion of penetrating disease and perianal disease, and a lower proportion of non-stricturing disease and ileal disease (Table 2).

**Table 2.** Patients with Crohn’s disease diagnosed in 2006–2013 versus those diagnosed in 2014–2020.

	2006–2013 (*n* = 56)	2014–2020 (*n* = 98)	*p*-Value
Mean age at diagnosis, years (SD)	34.0 (12.5)	35.8 (12.2)	0.387
Proportion of women (%)	33.9	52.0	0.030
Montreal classification (%)			
A2 (17–40 years)	67.9	66.3	0.846
A3 (40–65 years)	32.1	33.7	0.846
L1 (ileal)	25.0	43.9	0.027
L2 (colonic)	44.6	30.6	0.080
L3 (ileocolonic)	23.2	25.5	0.751
L4 (isolated upper disease)	7.2	0	0.016
B1 (non-stricturing, non-penetrating)	53.6	70.4	0.036
B2 (structuring)	17.9	24.5	0.340
B3 (penetrating)	28.6	5.1	<0.001
P (perianal disease)	17.9	5.1	0.010
Prednisolone in the first 5 years since diagnosis^a^			
Any prescription, %	66.1	26.1	<0.001
Median cumulative dose, mg (25th–75th percentile)	980 (0–1942)	0 (0–1337)	0.004
Proportion of patients with a cumulative prednisolone dose >1 g, % (*n* = 38/37)	45	38	0.544
Proportion of patients with a cumulative prednisolone dose >3 g, % (*n* = 38/37)	13	5	0.449
Budesonide in the first 5 years since diagnosis^a^			
Any prescription, %	51.8	68.1	0.063
Median cumulative dose, mg (25th–75th percentile)	91 (0–1282)	630 (0–1620)	0.145
Any prescription of rectal corticosteroids (%)	21.4	3.1	<0.001
Any prescription (%)			
5-ASA	57.1	28.6	<0.001
Thiopurines	75.0	67.3	0.318
TNF inhibitors	33.9	34.7	0.923
Vedolizumab	0	0	>0.999
Ustekinumab	0	3.0	0.295
Tofacitinib	0	1.0	>0.999
Surgery within the first 5 years since diagnosis^a^ (%)	26.8	8.6	0.002

a Includes only patients diagnosed 2006–2018.

5-ASA, five-acetyl salicylic acid.

### The use of prednisolone and budesonide in the first 5 years after diagnosis

The proportion of patients with IBD (UC + CD) who were prescribed at least one prescription of systemic corticosteroids (prednisolone or budesonide) in years 1–5 after diagnosis were 55.3%, 27.9%, 22.7%, 14.1%, and 14.6%, respectively. Patients with CD were significantly more often prescribed systemic corticosteroids in year 1 (81.9% vs 37.5%; *p* < 0.001), year 2 (33.5% vs24.2%; *p* = 0.034), and in year 5 (21.4% vs 10.3%; *p* = 0.007) after diagnosis compared to patients with UC (Table 3). Patients with CD were significantly more often prescribed systemic prednisolone (43.2% vs 34.5%, *p* = 0.035) in the first year after diagnosis but significantly less often in year 2 (10.3% vs 22.4%; *p* = 0.002), in year 3 (7.7% vs19.8%; *p* = 0.001), and in year 4 (4.1% vs 11.0%; *p* = 0.020) compared to patients with UC (Table 3). There were significantly more patients with UC than patients with CD who were prescribed a dosage >1 g systemic prednisolone in year 2 (13.4% vs 5.2%; *p* = 0.009), in year 3 (11.6% vs 2.6%; *p* = 0.001), in year 4 (6.4% vs 1.4%; *p* = 0.020), and in year 5 (5.4% vs 0%; *p* = 0.008) after diagnosis (Table 3).

**Table 3.** The use of systemic corticosteroids (prednisolone or budesonide) in the first 5 years after diagnosis in patients with ulcerative colitis and Crohn’s disease.

Ulcerative colitis	Year 1	Year 2	Year 3	Year 4	Year 5
	*N* = 232	*N* = 232	*N* = 232	*N* = 219	*N* = 203
The proportion of patients with at least one course of systemic corticosteroids (%)	37.5	24.2	20.2	11.5	10.3
The proportion of patients with at least one course of prednisolone (%)	34.5	22.4	19.8	11.0	8.9
The proportion of patients with a prescribed prednisolone dosage > 1 g (%)	23.3	13.4	11.6	6.4	5.4
Crohn’s disease	*N* = 155	*N* = 155	*N* = 155	*N* = 147	*N* = 128
The proportion of patients with at least one course of systemic corticosteroids, %	81.9	33.5	26.5	18.0	21.4
The proportion of patients with at least one course of prednisolone (%)	43.2	10.3	7.7	4.1	7.0
The proportion of patients with a prescribed prednisolone dosage >1 g (%)	25.8	5.2	2.6	1.4	0

The proportion of patients who did not receive any prescription of corticosteroids in the first 5 years after diagnosis was 34.3% for all patients with IBD, 46.3% for UC patients, and 15.1% for CD patients. The proportion of patients who did not receive any prescription for prednisolone the first 5 years after diagnosis was 49.8% for all patients with IBD 49.3% for UC patients, and 50.1% for CD patients.

### Time trends in the prescription of corticosteroids for 2006–2023

**Figure 2.**
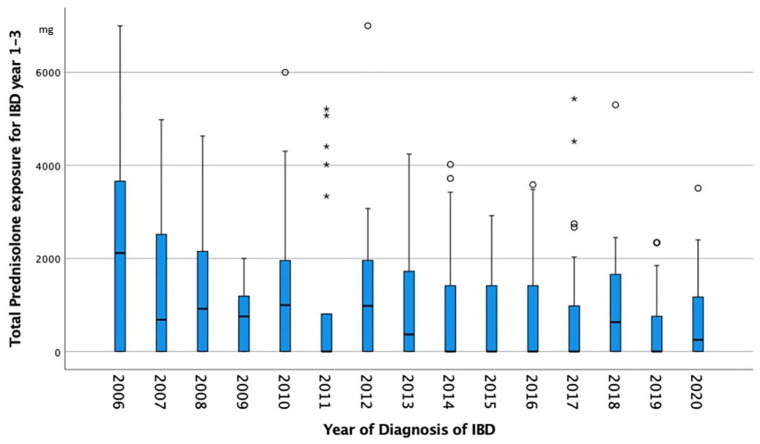
There was a significant decline in the cumulative prescribed prednisolone dosage in the first 3 years after diagnosis for patients with IBD diagnosed between 2006 and 2020 (rs = −0.164; *p* = 0.001). The black line indicates the median prescribed dosage and the bars the 75th percentile. Circles and asterisks show outliers. IBD, inflammatory bowel disease.

There was a decline in the cumulative prescribed systemic dosage of prednisolone (UC: rs = −0.111, *p* = 0.092 and CD rs = −0.246, *p* = 0.002) (Figure 2) and an increase in the cumulative prescribed dose of budesonide (UC: rs = 0.158, *p* = 0.016 and CD rs = 0.130, *p* = 0.107) in the first 3 years after diagnosis.

**Figure 3.**
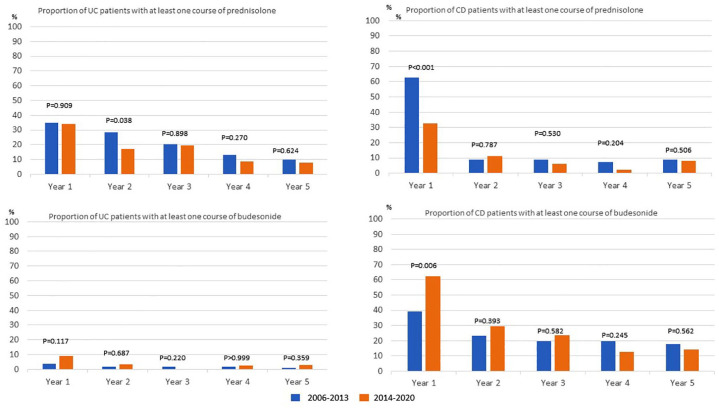
Patients with UC and CD diagnosed in 2006–2013 versus 2014–2020, and the proportion of patients that at least once received a prescription of prednisolone or budesonide in the first 5 years after diagnosis. CD, Crohn’s disease; UC, ulcerative colitis.

Being prescribed at least one course of prednisolone in the first year of diagnosis was significantly more common among patients with CD diagnosed in 2006–2013, whereas being prescribed at least one course of budesonide in the first year after diagnosis was significantly more common among patients with CD diagnosed in 2014–2020 (Figure 3). Overall, there were no differences in the proportion of patients with UC who received at least one prescription of prednisolone and budesonide in the first 5 years after diagnosis between the two time periods; an exception was for the second year after diagnosis, in which patients who were diagnosed 2014–2020 had a significantly lower extent of prescription of systemic prednisolone (Figure 3).

The proportion of patients who were prescribed rectal steroid treatment was significantly higher in patients with UC and CD diagnosed in 2006–2013 compared to patients diagnosed in 2014–2020 (Tables 1 and 2).

### Time trends in the prescription of other medical treatments for 2006–2023

**Figure 4.**
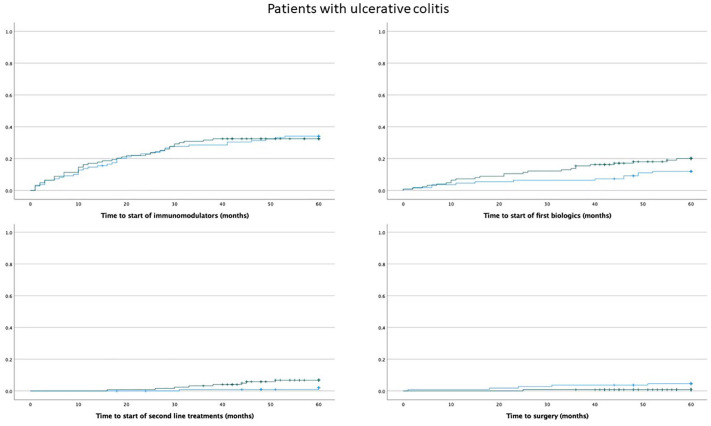
Kaplan-Meier curves showing the time to the first prescription of immunomodulators, biologics (TNF inhibitors), second-line treatment (vedolizumab, ustekinumab, or tofacitinib), and the time to first surgery after diagnosis in patients with UC. The blue line represents patients diagnosed in 2006–2013 and the green line represents patients diagnosed in 2014–2020. UC, ulcerative colitis.

**Figure 5.**
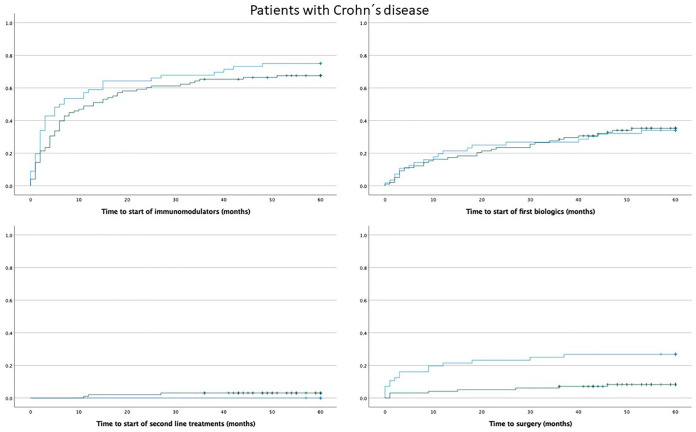
Kaplan-Meier curves showing the time to the first prescription of immunomodulators, biologics (TNF inhibitors), second-line treatment (vedolizumab, ustekinumab, or tofacitinib), and the time to first surgery after diagnosis in patients with Crohn’s disease. The blue line represents patients diagnosed in 2006–2013 and the green line represents patients diagnosed in 2014–2020.

The prescription of any immunomodulator in the first 5 years from diagnosis was stable in the years 2006–2018 (rs = 0.056; *p* = 0.257), but there was a slight increase in the prescription of TNF inhibitors (rs = 0.119, *p* = 0.020) in patients with IBD. There were no significant differences in the time to start immunomodulators between patients diagnosed with UC or CD diagnosed 2006–2013 versus those diagnosed 2014–2020 (Mantel–Cox test: UC, 0.902, and CD, *p* = 0.202) (Figures 4 and 5). Patients with UC diagnosed 2014–2020 started TNF-inhibitor treatment earlier than patients diagnosed 2006–2013, but the difference was not statistically significant (Mantel–Cox test *p* = 0.091) (Figure 4). The time to start TNF inhibitors was similar in patients with CD (Mantel–Cox test, *p* = 0.937) (Figure 5).

The proportion of patients who were prescribed second-line therapy (vedolizumab, ustekinumab, and tofacitinib) was 3.4% for all included IBD patients. The time to start second-line treatment in patients with IBD was significantly shorter in those diagnosed in 2014–2020 versus 2006–2013 (Mantel–Cox test, *p* = 0.036).

There was a significant decline in the proportion of patients with CD (rs = −201; *p* = 0.012) and in the patients with IBD (rs = −0.172, *p* < 0.001) who were prescribed 5-ASA between 2006 and 2023 (Tables 2 and 3), but the prescriptions for patients with UC were stable (rs = 0.045, *p* = 0.496) (Table 1).

### The patients with at least 1 year of a prescribed prednisolone dosage >1 g

For all patients with IBD, 132 patients (34.2%) had at least 1 year with cumulative prescribed systemic prednisolone dosage >1 g in the first 5 years after diagnosis (37.7% UC and 28.6% CD). Among these patients, 35 patients with CD (80.0%) and 47 patients with UC (53.4%) received a corticosteroid-sparing agent (immunomodulator and/or biologic) according to guidelines. For the corticosteroid-sparing agents 40 patients were prescribed immunomodulators and 42 patients were prescribed combination therapy (immunomodulators and biologics). There were no differences between the proportion of patients who received a corticosteroid-sparing agent between 2006 and 2013 versus 2014 and 2020 (62.9% vs 61.5%; *p* = 0.892). The median cumulative systemic prescribed prednisolone dosage (year 1–5 after diagnosis) in the patients who did not receive a corticosteroid-sparing agent was 2340 mg (25th–75th percentile 1415–3720 mg), and this did not differ from those who did receive a corticosteroid-sparing agent (median 2340 mg; 25th–75th percentile 1715–3510 mg). There were no differences in the prescribed dosage of systemic prednisolone on an annual basis (year 1, 2, 3, 4, or 5 after diagnosis) in those who received a corticosteroid-sparing agent versus those who did not.

### Time trends in surgery for 2006–2023

There was a significant decline in the proportion of patients with IBD who underwent surgery within the first 5 years after diagnosis in patients diagnosed 2006–2018 (rs = −0.164, *p* = 0.001). The proportion of patients with UC who were colectomized within the first 5 years of diagnosis was lower in patients diagnosed in 2014–2018 compared to those diagnosed in 2006–2013, but the difference was not statistically significant (Table 1). Only one patient diagnosed with UC in 2014–2018 was colectomized within the first 5 years after diagnosis.

There was a significant decline in the proportion of patients with CD who underwent surgery within the first 5 years after diagnosis between those diagnosed in 2014 and 2018 versus those diagnosed 2006 and 2013 (Table 2). The time to surgery in patients diagnosed 2014–2020 was significantly shorter than those diagnosed 2006–2013 (Mantel–Cox test, *p* = 0.001).

## Discussion

The present study determined the extent to which patients with IBD were prescribed corticosteroids for the first 5 years after diagnosis. Two of three patients with IBD received at least one prescription of systemic corticosteroids (prednisolone or budesonide), and half of the patients with IBD were prescribed systemic prednisolone at least once in the first 5 years after diagnosis. However, the proportion of patients with IBD who received a prescription for prednisolone at our clinic decreased beyond the first year after diagnosis, and in the second to fifth year after diagnosis, the proportion was relatively low. For example, in patients with UC, approximately 10% had systemic prednisolone prescribed in years 4 and 5 and the proportion was even lower in patients with CD.

A daily dosage of prednisolone ⩾ 15 mg for 2–3 months and a cumulative dosage >1 g have been associated with an increased risk of hip fractures, and exceeding this dosage has been suggested as a reason to start therapeutic intervention to prevent fractures.^10^ Approximately 38% of our patients with UC and 29% of the patients with CD had at least 1 year with a prescription of systemic prednisolone that exceeded 1 g. In our study, prednisolone was mostly prescribed in the first year, but even shorter courses of high-dose prednisolone were shown to be associated with fracture risk.^10^ We did not measure the length of treatment but instead measured the cumulative prescribed dose. In a study from the United Kingdom of patients with IBD treated in primary care and diagnosed between 2002 and 2005, approximately 27% with CD and 14% with UC received a prolonged steroid exposure (>3 months) at least once in the first 5 years after diagnosis.^20^

When analyzing the cumulative prescribed prednisolone dose in the first 3 years after diagnosis, our study showed a minor decline in the prescription of both oral and rectal prednisolone treatment between 2006 and 2023, but an increase in the use of oral budesonide, especially in patients with CD. The decrease in prednisolone use is consistent with studies from, Denmark,^17^ the Netherlands,^18^ Israel,^19^ and the United States.^22^ A study from primary care showed that the use of “any steroid” and budesonide had increased, but prolonged steroid use had declined.^20^ Also, Targownik et al.^22^ showed increased use of budesonide in CD patients in the United States between 2006 and 2017. For patients with UC at our hospital, there was only a modest decrease in the use of systemic prednisolone during the study period, which is a pattern also seen in the United States.^22^ In the literature, there are sparse data on the time trends for topical corticosteroid therapy, and the cause of the decline in the prescription of rectal corticosteroids seen in our study is difficult to explain but may be due to the introduction of novel therapies.

In 2014, the introduction of biosimilars remarkably reduced the cost of TNF inhibitors.^13^ In our study, we observed a slight increase in the prescription of TNF inhibitors after 2014, which partly explains the reduction in prednisolone prescription. In addition, especially in patients with CD, the increased prescription of budesonide may contribute to the lower use of prednisolone. However, in a Canadian study, corticosteroid prescriptions had not changed despite the increased use of biologics.^21^

Budesonide has been associated with fewer side effects than prednisolone,^27^ and when used in maintenance therapy (1 year), adverse events did not differ from placebo.^28^ Contrary to patients treated with prednisolone,^10,29,30^ patients treated with budesonide probably have lower and insignificant risks for osteoporosis^31,32^ and adrenal insufficiency.^28,33^

Compared to other studies, our clinic had a lower prescription of biologics than expected. For example, in a National French study of patients diagnosed in 2009–2013, the 5-year cumulative probability of receiving treatment with TNF inhibitors was 49% for patients with CD and 20% for patients with UC.^34^ In our study, the 5-year cumulative probability of receiving TNF inhibitors was 34% for CD and 12% for UC. Additionally, the prescription of vedoluzimab, uteskinumab, and tofacitinib was lower than expected.

Furthermore, the proportion of patients who received a corticosteroid-sparing agent according to guidelines was 80% for the patients with CD but only about 50% for patients with UC. But interestingly, we found no differences in the cumulative prescription of systemic prednisolone between those patients who received a corticosteroid-sparing agent and those who did not.

### Limitations

A limitation is that the study only had data on the prescription of corticosteroids but lacked data on the use of corticosteroids (whether or not the patient went to the pharmacy and complied with the prescription). Furthermore, we did not present data on corticosteroid prescription beyond 5 years after diagnosis or in patients diagnosed at elderly ages (>65 years). Finally, the overall sample size is relatively small and could affect the results when comparing subcategories with low numbers.

A strength of our study is that our medical record system allows us to receive data on all prescriptions made in the entire Region of Västerbotten. Another strength is that we present data on the cumulative dosage prescribed.

## Conclusion

In conclusion, approximately 38% of the patients with UC and 29% of the patients with CD had at least 1 year with a prescription of systemic prednisolone that exceeded 1 g. There was a decrease in prescription of prednisolone and an increase in the prescription of budesonide treatment from 2006 to 2023, but the cumulative exposure to corticosteroids in patients with IBD remained at a relatively high level.

## Supplemental Material

sj-docx-1-tag-10.1177_17562848241288851 – Supplemental material for Prescribed cumulative dosage of corticosteroids to patients with inflammatory bowel disease diagnosed between 2006 and 2020: a retrospective observational study Supplemental material, sj-docx-1-tag-10.1177_17562848241288851 for Prescribed cumulative dosage of corticosteroids to patients with inflammatory bowel disease diagnosed between 2006 and 2020: a retrospective observational study by Johannes Iiristo and Pontus Karling in Therapeutic Advances in Gastroenterology

## Declarations

**Ethics approval and consent to participate:** The study was reviewed by the Swedish National Review Board (Dnr 2022-060609-01) and was judged to have no significant ethical considerations due to its non-interventional design. The study was performed in accordance with Swedish law for Patient Safety and the directions of the Swedish Data Protection Authority. Due to the retrospective observational design of the study and because the study evaluated clinical practice there was no consent obtained from the patients.

**Consent for publication:** Not applicable.

**Availability of data and materials:** Selected data are available from the corresponding author on a reasonable request.
